# Are the Goals of Therapy Achievable in Patients with Rheumatoid Arthritis Receiving Upadacitinib in Real Clinical Practice?

**DOI:** 10.1134/S1607672923700308

**Published:** 2023-10-13

**Authors:** V. N. Amirdzhanova, A. E. Karateev, E. Yu. Pogozheva, E. S. Filatova, R. R. Samigullina, V. I. Mazurov, O. N. Anoshenkova, N. A. Lapkina, A. A. Baranov, T. Yu. Grineva, A. M. Lila, E. L. Nasonov

**Affiliations:** 1 Nasonova Research Institute of Rheumatology, Moscow, Russia; 2 Sechenov First Moscow State Medical University, Ministry of Health of the Russian Federation (Sechenov University), Moscow, Russia; 3 Russian Medical Academy of Continuing Professional Education, Ministry of Health of the Russian Federation, Moscow, Russia; 4 Mechnikov Northwestern State Medical University, Ministry of Health of the Russian Federation, St. Petersburg, Russia; 5 Medical Center “Maximum Health”, Tomsk, Russia; 6 Yaroslavl State Medical University, Ministry of Health of the Russian Federation, Yaroslavl, Russia; 7 Vologda Regional Clinical Hospital No. 1, Vologda, Russia

**Keywords:** upadacitinib, rheumatoid arthritis, remission

## Abstract

The aim of the study was to evaluate the effectiveness of UPA in RA patients in real clinical practice after 3 and 6 months of therapy. The study included 63 RA patients with high activity of the disease. Activity was assessed according to the DAS28(ESR), DAS28(CRP), SDAI, CDAI; functional ability to HAQ; quality of life to the EQ-5D; disease activity according to the patient’s RAPID-3 index; the level of depression and anxiety to the HADS scale. The effectiveness of therapy was evaluated after 3 (*n* = 45) and 6 (*n* = 31) months of UPA therapy. Remission or low activity of the disease by 3 months of therapy was achieved by most patients: remission of 69.8% of patients, low activity of the disease—16.3% of patients. Moderate or high activity persisted in 13.9% of patients. By the 6th month of UPA therapy, the number of remissions reached 90%, low activity 3.3%, moderate activity persisted in 6.7% of patients, high activity of the disease was not in any patient. 20% improvement in function was achieved in 71.8% of patients by the 3rd month of therapy and in 77.8% by the 6th month of treatment; the difference in average HAQ values by the 3rd month of therapy was 0.38 points, by the 6th month—0.58 points. After 3 months of follow—up, 31.1% of patients continued taking GC, by 6 months—24.2%. The dose of GC was reduced from an average of 7.23 to 5.6 mg/s. The percentage of patients requiring NSAIDs decreased from 95.2 to 35.6% and 33.3%, respectively. DMARDs continued to be received by 75.6% of patients by 3 months and 69.7% by 6 months of follow-up. Achieving remission or low activity of the disease in patients with RA receiving UPA in real clinical practice is possible in most patients. A rapid decrease in inflammatory activity is accompanied by a significant improvement in the functional state and quality of life of patients. UPA therapy reduces the need for the use of NSAIDs and reduces the dose of GC in a third of patients.

An integral part of the “Treat to target” strategy in the management of patients with rheumatoid arthritis (RA) is the achievement of remission or at least low disease activity. Therapy for RA usually begins with the use of disease-modifying antirheumatic drugs (DMARDs) with or without low doses of glucocorticoids (GCs). If they are ineffective or intolerable in therapeutic doses, genetically engineered biological drugs (biologics) or targeted synthetic DMARDs can be used as a first-line therapy [1]. However, even if the recommendations for managing patients treated with biologics are followed, in real clinical practice remission is achieved only in 20–40% of cases. Therefore, in some patients, optimal outcomes of the disease are not observed [2]. Expanding knowledge about the mechanisms of RA pathogenesis stimulated the development of a wide range of novel synthetic targeted DMARDs [3–5]. In this series, a special place is occupied by Janus kinase (JAK) inhibitors [6–9], the introduction of which into clinical practice has significantly expanded the opportunities of RA pharmacotherapy.

Upadacitinib (UPA), a selective JAK-1 inhibitor, is a new therapeutic option, the use of which for the treatment of patients with rheumatoid arthritis (RA) according to international randomized clinical trials (RCTs) [10–16] allows patients with insufficient effect of therapy with DMARDs or biologics achieve therapy goals fairly quickly. However, currently, information on whether remission in RA patients receiving the drug can be achieved in real clinical practice with long-term follow-up is insufficient.

The aim of this study was to evaluate the UPA efficacy in RA patients in real clinical practice after 3 and 6 months of therapy.

## MATERIALS AND METHODS

The Russian project RAKURS [17] included 63 RA patients. Their treatment with UPA was initiated in seven rheumatological centers of the Russian Federation. The criteria for inclusion of patients in the project were as follows: age over 18 years; confirmed diagnosis of RA according to ACR/EULAR 2010 criteria; moderate or high RA activity according to the DAS28 index; intolerance or ineffectiveness of previous therapy with methotrexate (MT), other DMARDs, or biologics for at least 6 months; patient consent to participate in the study (signed informed consent). Exclusion criteria were as follows: planning pregnancy and breastfeeding period; patients with active severe infection, including localized infections, and with contraindications for initiating UPA therapy. UPA therapy was prescribed in accordance with the indications approved in the instructions for medical use of the drug, as well as the decision of the attending physician and commission on therapy with biologics.

During the observation, data were collected from the disease history, previous therapy, and concomitant diseases. When initiating therapy, information about the absence of tuberculosis was taken into account, and a PCR test for COVID-19 was performed (no more than 5 days from the initiation of therapy). The therapy with DMARDs, GCs, biologics, and NSAIDs was noted. Every 3 months, a complete clinical examination of the patient was carried out with an assessment of the duration of morning stiffness in minutes and its intensity on a visual analog scale (VAS), the number of painful (NPJ) and swollen (NSJ) joints was counted, the general health status was assessed by the doctor (GHSD) and the patient (GHSP), the level of pain and fatigue was assessed by VAS, ESR was determined according to Westergren, and CRP (mg/L and mg/dL) was determined. General clinical, biochemical and immunological blood tests and urinalysis were performed. When initiating therapy, chest radiography (or CT scan) and ECG were taken.

To assess the activity of the disease, the standard indices DAS28ESR, DAS28CRP, SDAI, and CDAI were used. Functional ability was assessed by the HAQ index. Changes of more than 0.22 points were considered minimal clinically significant changes in the functional state of patients according to the HAQ index. The percentage of patients with a population value of the HAQ index ≤0.5 points was calculated. Quality of life (QoL) was assessed using the EQ-5D questionnaire. The disease activity according to the patient was assessed using the RAPID-3 index: high disease activity corresponded to index values of more than 12 points; moderate activity, from 6.1 to 12 points; low activity, from 3.1 to 6.0 points; and remission, 3 points and below.

To reveal the states of depression, anxiety, and emotional distress, the HADS scale was used [18, 19]. Separate scoring was carried out independently for the questions assessing the level of depression and for the questions assessing the degree of anxiety. To interpret the results, the total score for each scale was used. A score from 0 to 7 points was considered a normal value, subclinically expressed anxiety/depression was determined at values from 8 to 10 points, and clinically expressed symptoms of depression or anxiety were detected at 11 points and above.

The effectiveness of therapy was evaluated according to the dynamics of disease activity indices after 3 (*n* = 45) and 6 (*n* = 31) months of UPA therapy.

The analysis of patient data is based on the Electronic Database of Patients with Rheumatoid Arthritis, created by RSMI LLC using its own online platform. Data were statistically processed using the Microsoft Excel software and the Statistica 10 for Windows statistical data analysis package (StatSoft Inc., United States) using the generally accepted methods of parametric and nonparametric analysis. Differences were considered statistically significant at *p* < 0.05.

## RESULTS AND DISCUSSION

The majority of patients (84%) were female, 54% continued to work, and 16% were retired. Almost half of the patients did not have a disability (46%) or were partially able to work with a group III disability (33.3%), 17.5% had group II disability, and 3.2% of patients had group I disability. Functional insufficiency of functional classes II and III was observed in 93.6% of patients.

The duration of the disease was, on average, 10.6 ± 9.3 years. Extra-articular manifestations of the disease were detected in 22.2% of patients in the form of rheumatoid nodules (*n* = 11), Sjögren’s syndrome (*n* = 2), and polyneuropathy (*n* = 2). The age of patients at the initiation of therapy was 53.9 ± 14.3 years, the body mass index was 26.0 ± 4.6. Most patients were seropositive for rheumatoid factor (87.3%) and ACCP (79.4%), in 81% of cases they had comorbid pathology with prevailing diseases of the gastrointestinal tract (54%), arterial hypertension (41.3%), cardiovascular pathology (3.2%), diseases of the liver and gallbladder (27%), diabetes mellitus (1.6%), and varicose veins (7.9%). One comorbidity was found in 30.8% of patients, two in 13.5%, three in 25%, and four in 13.5% of patients. Only 17.2% of patients were without comorbid pathology.

Endoprosthetics of the knee or hip joints was previously performed in 12.6% of patients, some patients underwent surgery on the hands and feet (3.2%).

Despite the current recommendations for vaccination of RA patients, only 8% of patients were vaccinated against influenza, 14.3% against pneumococcus, and 12.7% against COVID-19.

Nine patients (14.3%) had a coronavirus infection: one was asymptomatic, four were in a mild form at home, three were in a moderate form in a hospital, and one was in a severe form with 75% lung damage. In these patients, UPA was discontinued for the duration of the viral infection, and all patients resumed the drug after recovery.

Before the initiation of UPA therapy, the majority of patients (52.4%) received methotrexate (MT) (pill form in 15.9% of patients at an average dose of 10.75 ± 2.5 mg per week). The duration of MT intake was 61.2 months. The impossibility of increasing the dose of MT was due to poor tolerability of this drug. The subcutaneous form of MT was received by 36.5% of patients, the dose of the drug when administered subcutaneously was significantly higher compared to the MT pills (19.5 ± 4.4 mg/week (*p* <0.01)); the average duration of intake was 48.4 months.

At insufficient efficacy of MT, given the impossibility of increasing its dose due to adverse reactions or poor tolerance, 22.2% of patients received leflunomide for the entire duration of the disease; the average duration of treatment was 28.8 months.

Hydroxychloroquine was received by three patients (4.8%).

Due to the high activity of the disease, 65.1% of patients before initiating UPA therapy were on glucocorticoid (GC) therapy at an average dose of 6.8 ± 2.4 mg/day in terms of prednisolone. All patients had to take NSAIDs: 46.6% received coxibs, 23.3% received nimesulides or meloxicams, and 30.1% received other NSAIDs on a regular basis.

Before the prescription of UPA, 49 patients (77.8%) received biologicals, mainly TNF-a inhibitors (42.8%) (2-etanercept, 4-adalimumab, 10-infliximab, 3-golimumab, and 2-certolizumab-pegol); 17 patients (34.7%) received rituximab; five patients (10.2%) received interleukin-6 receptor inhibitors (tocilizumab, levilimab, and sarilumab); four patients (8.2%) received abatacept, and two patients (4.1%) received tofacitinib. The duration of intake of biologics varied from 6 to 48 months. The patients previously treated with biologics did not differ in the main parameters from the patients who received only DMARDs and GCs. However, they had a longer duration of the disease (16.2 ± 3.2 compared to 5.6 ± 1.8 years, *p* < 0.001), a lower ESR (23 ± 2.1 and 35.7 ± 2.3, *p* < 0.001), and disease activity according to DAS 28 CRP (4.9 ± 0.5 and 5.4 ± 1.2, *p* < 0.045). Patients who needed therapy with biologics received a higher weekly dose of MT (17.0 ± 1.2 and 9.0 ± 1.5, respectively; *p* < 0.018).

Before the initiation of UPA therapy, the duration of morning stiffness was 147.4 ± 228.0 min, its severity according to VAS averaged 51.3 ± 31.4 mm; the number of painful joints was 10.1 ± 5.8; the number of swollen joints was 7.0 ± 4.4; the general health status according to the patient (GSHP) was 60.9 ± 17.4; the general health status according to the doctor (GSHD) was 57.2 ± 14.9.

The mean values of the standard indices corresponded to moderate or high disease activity: DAS28 (CRP) 5.2 ± 1.0; DAS28 (ESR) 5.5 ± 1.1; SDAI 51.2 ± 32.4; CDAI 28.9 ± 19.5. Pain severity averaged 56.8 ± 25.8 mm according to VAS and correlated with DAS28 activity indices (ESR) *r* = 0.355 (*p* = 0.005); DAS28 (CRP) *r* = 0.303 (*p* = 0.017); quality of life according to EQ-5D *r* = –0.29 (*p* = 0.020) and GHSP *r* = 0.26 (*p* = 0.043).

The mean values of the functional state of RA patients according to the HAQ index were 1.9 ± 1.1; there were no population-based HAQ values in any patient.

The mean function values according to the RAPID-3 questionnaire in RA patients were 4.4 ± 2.3 points; pain 6.2 ± 1.8; general health status 6.3 ± 1.8. The calculated total value of the three scales, representing the RAPID-3 index, averaged 16.8 ± 5.0 points, which, according to the patients, confirmed that they had a high disease activity.

The quality of life of RA patients according to the general questionnaire EQ-5D was low (0.52 [–0.59; 0.8]). The level of anxiety on the HADS scale averaged 9.6 ± 6.3 points, depression averaged 8.0 ± 5.1 points, which corresponded to subclinically expressed symptoms of anxiety and depression.

After 3 (*n* = 45) and 6 (*n* = 31) months of UPA therapy, there was a significant (*p* < 0.001) decrease in all clinical indices: morning stiffness, pain, number of painful and swollen joints, ESR and CRP levels, and disease activity indices (DAS28 ESR and CRP, CDAI, CDAI). In general, fatigue in patients decreased, the level of anxiety and depression also decreased, and sleep and functional state improved according to the HAQ and RAPID-3 indices (Tables 1, 2).

**Table 1.** Dynamics of clinical indices of RA patients receiving UPA (*p* <0.001 compared with initiation of therapy)

	Duration of morning stiffness (min)	Severity of morning stiffness (VAS)	Number of painful joints	Number of swollen joints	General assessment of the patient’s state of health (VAS)	General health assessment by a doctor (VAS)	ESR(mm/h)	CRP(mg/L)	Pain (VAS)
Initiation of therapy (*n* = 63)	90.0 ± 27.2	50.0 ± 11.0	9.0 ± 2.0	7.2 ± 1.3	67.0 ± 13.2	60.0 ± 11.2	26.0 ± 13.4	15.0 ± 2.6	60.0 ± 5.3
3 months (*n* = 45)	10.0 ± 2.2	5.0 ± 0.8	3.0 ± 1.2	1.0 ± 0.5	30.0 ± 5.4	30.0 ± 4.3	19.0 ± 2.1	2.25 ± 0.5	30.0 ± 3.5
6 months (*n* = 31)	15.0 ± 3.5	3.0 ± 0.5	2.0 ± 0.5	0.5 ± 0.3	30.0 ± 2.1	30.0 ± 2.2	15.0 ± 1.7	2.0 ± 0.5	20.0 ± 4.3

**Table 2.** Dynamics of activity indices and functional status of RA patients receiving UPA (*p* < 0.001 compared with initiation of therapy)

	DAS28 (CRP)	SDAI	CDAI	HAQ	RAPID-3	HADS (anxiety)	HADS (depression)	Ftigue	Sleep
Initiation of therapy (*n* = 63)	5.2 ± 1.1	51.2 ± 32.4	28.9 ± 10.5	1.9 ± 1.1	17.0 ± 2.1	9.6 ± 6.3	8.0 ± 5.1	5.8 ± 1.1	1.7 ± 0.2
3 months (*n* = 45)	3.0 ± 0.4	13.1 ± 10.2	10.0 ± 5.1	1.0 ± 0.3	8.0 ± 1.8	4.5 ± 1.1	3.5 ± 1.2	3.0 ± 1.2	1.0 ± 0.4
6 months (*n* = 31)	2.0* ± 0.3	12.6 ± 8.1	9.3 ± 4.2	0.8 ± 0.2	7.0 ± 0.3	3.5** ± 1.1	3.0*** ± 1.2	3.0 ± 1.1	0.7 ± 0.2

*p = 0.024, **p = 0.029, ***p = 0.021.

By 3 months of therapy, 28.2% of patients did not notice minimal clinically significant changes in their functional state, by 6 months of therapy their number decreased to 22.2% (Table 3). At least 20% improvement in function according to patients was achieved in  71.8% of patients by 3 months and in 77.8% by 6 months of UPA treatment.

**Table 3.** Dynamics of the functional state of patients according to the HAQ index

∆HAQ (points)		3 months (*n* = 45)		6 months (*n* = 31)	
		*n*	%	*n*	%
∆HAQ < 0.22	No effect	11	28.2	6	22.2
0.22 ≤ ∆HAQ ≤ 0.36	Improvement	1	2.6	1	3.7
0.36 < ∆HAQ < 0.80	Improvement	13	33.3	6	22.2
∆HAQ ≥ 0.80	Improvement	14	35.9	14	51.9

A 50% improvement was achieved in one-third of patients, and a pronounced improvement in the functional state (70% improvement) was observed in 35.9% of patients by 3 months of therapy and in half of patients (51.9%) by 6 months of therapy.

The difference in the mean HAQ values was 0.38 points by 3 months of therapy and 0.58 points by 6 months. QoL according to the EQ-5D index improved in 98.5% of patients, 70% improvement was noted in more than one-third of them (41.7%).

The main goals of therapy (remission or low disease activity according to the DAS 28 (ESR) index by 3 months of therapy) were achieved by the majority of patients: remission in 69.8% of patients, low disease activity in 16.3%. Moderate or high disease activity remained in 13.9% of patients.

It should be noted that, at the initiation of UPA therapy, the patients who subsequently achieved remission or low disease activity did not differ in basic characteristics from the patients with worse treatment outcomes in terms of age, disease duration, doses and duration of DMARDs and biologics, pain, fatigue, levels of anxiety and depression, and QoL according to EQ-5D (*p* > 0.05). Patients who did not achieve remission by 3 and 6 months of UPA therapy initially had more pronounced morning stiffness (330.0 ± 307.0 min and 142.9 ± 146.2 min, respectively; *p* < 0.033), a high CRP level (31.9 ± 3.7 and 21.9 ± 31.2, respectively; *p* < 0.024), and higher disease activity indices DAS28 (6.0 ± 0.8 and 5.4 ± 1.0, respectively; *p* < 0.03), CDAI (37.0 ± 8.9 and 27.7 ± 9.1, respectively; *p* < 0.036), and SDAI (68.9 ± 18.8 and 49.6 ± 37.2, respectively; *p* < 0.012).

By 6 months of therapy, the number of remissions increased to 90%, low activity remained in 3.3%, moderate activity remained in 6.7% of patients, and no patient had high disease activity. The evaluation of the results of therapy according to the DAS28 (CRP) index was more modest: remission and low activity were registered in 69.7% by 3 months of therapy and in 46.7% by 6 months of observation; disease activity remained high in 4.7 and 13.3% of patients, respectively.

At the initiation of therapy, 65.1% of patients received GCs. By 3 months of therapy, 31.1% of patients remained on GC; by 6 months, 24.2%. The GC dose was reduced from an average of 7.23 to 5.6 mg/s. The percentage of patients requiring NSAIDs decreased from 95.2 to 35.6% and 33.3%, respectively. As many as 75.6 and 69.7% of patients continued to receive DMARDs by 3 and 6 months of follow-up.

The activity of the disease in the dynamics according to the patient was assessed by the combined RAPID-3 index. At the initiation of therapy, the average value of the index was 16.8 ± 5.0 points, which corresponded to the definition of “high disease activity.” After 3 and 6 months of therapy, patients noted a significant decrease to 8.0 ± 1.2 and 7.0 ± 0.6, respectively (*p* < 0.001). The therapy was well tolerated by all patients, and no serious adverse events were recorded.

Can we now talk about a shift in the paradigm of treatment of RA with the use of JAK inhibitors, or is it just an addition of another group of effective drugs for the treatment of immune-inflammatory joint diseases? Today, when insufficient time has passed to assess the long-term results of their long-term use, it is very difficult to answer this question, since the effectiveness of therapy, including the achievement of remission and low disease activity, with the use of TNF-alpha inhibitors and JAK inhibitors practically coincide.

Currently, there is great interest in the study of the new selective JAK-1 inhibitor, UPA (Ranvek), for the treatment of RA patients in real clinical practice, especially in the patients who are difficult to treat [20]. UPA, unlike biologics, is intended for oral administration and does not cause generation of antibodies that can neutralize the activity of protein molecules and thereby reduce the effectiveness of treatment, and in the future it may contribute to a change in the paradigm of pharmacotherapy of this disease. Its efficacy and safety, previously proven in RCTs [10–16], which are comparable to those of biologics, the convenience of use in the pill form, the absence of a “cold chain” during storage, and the possibility of rapid withdrawal and elimination of the drug in case of adverse events in some cases make it indispensable for some patients. The pronounced, rapid analgesic effect of UPA, which is significantly superior to all available biologics, is also well known.

A study of the efficacy and safety of UPA in real clinical practice conducted in seven centers of the Russian Federation showed that, after 3 and 6 months of UPA therapy, a significant (*p* < 0.001) decrease in all clinical indices (morning stiffness, pain, number of painful and swollen joints, ESR and CRP levels, and disease activity indices (DAS28 ESR and CRP, CDAI, and CDAI)) was detected. Other changes included a decrease in fatigue and in the levels of anxiety and depression, as well as an improvement in sleep and functional status assessed by HAQ and RAPID-3 indices. Remission or low disease activity according to the DAS 28 index (ESR) by the 3rd month of therapy was achieved in the majority of patients (remission in 69.8% and low disease activity in 16.3% of patients). Moderate or high disease activity was observed in 13.9% of patients. By the 6th month of therapy, the number of remissions reached 90%; low and moderate activity was observed in 3.3 and 6.7% of patients, respectively; and high disease activity was not detected in any patient. In addition, a 20% improvement in function was detected in 71.8% of patients by 3 months of therapy and in 77.8% of patients by 6 months of treatment; the difference in mean HAQ values was 0.38 points by 3 months and 0.58 points by 6 months of therapy, which indicates an improvement in the functional state of the majority of patients. Of great interest are data on the possibility of reducing the dose of GCs in UPA therapy. For example, after 3 months of observation, 31.1% of our patients continued to take GCs; by 6 months, 24.2%. The GC dose was reduced from an average of 7.23 to 5.6 mg/s. The percentage of patients requiring NSAIDs decreased from 95.2 to 35.6% and 33.3%, respectively. As much as 75.6% of patients continued receiving DMARDs at 3 months and 69.7% at 6 months of follow-up. Indeed, the development of targeted selective therapies with low-molecular-weight drugs, such as JAK-1 inhibitors, with different selective inhibitory profiles, in some cases made it possible to either completely cancel or reduce the dose of GCs, which was demonstrated in our study.

Thus, achieving remission or low disease activity in RA patients receiving UPA in clinical practice is a realistic goal for most patients. A rapid decrease in inflammatory activity and pain is accompanied by a significant improvement in the functional state and quality of life of patients. UPA therapy can reduce the need for NSAIDs and reduce the dose of GCs in a third of patients. The first results of the use of UPA in RA patients with insufficient efficacy of previous therapy with DMARDs or biologics in real clinical practice confirm the results on its efficacy and safety by 12 and 24 weeks of studies obtained in international clinical trials.
