# Erratum to: **Are the Goals of Therapy Achievable in Patients with Rheumatoid Arthritis Receiving Upadacitinib in Real Clinical Practice?**

**DOI:** 10.1134/S1607672923050010

**Published:** 2024-01-24

**Authors:** V. N. Amirdzhanova, A. E. Karateev, E. Yu. Pogozheva, E. S. Filatova, R. R. Samigullina, V. I. Mazurov, O. N. Anoshenkova, N. A. Lapkina, A. A. Baranov, T. Yu. Grineva, A. M. Lila, E. L. Nasonov

**Affiliations:** 1 Nasonova Research Institute of Rheumatology, Moscow, Russia; 2 Sechenov First Moscow State Medical University, Ministry of Health of the Russian Federation (Sechenov University), Moscow, Russia; 3 Russian Medical Academy of Continuing Professional Education, Ministry of Health of the Russian Federation, Moscow, Russia; 4 Mechnikov Northwestern State Medical University, Ministry of Health of the Russian Federation, St. Petersburg, Russia; 5 Medical Center “Maximum Health”, Tomsk, Russia; 6 Yaroslavl State Medical University, Ministry of Health of the Russian Federation, Yaroslavl, Russia; 7 Vologda Regional Clinical Hospital No. 1, Vologda, Russia

The article “Are the Goals of Therapy Achievable in Patients with Rheumatoid Arthritis Receiving Upadacitinib in Real Clinical Practice?”, written by V.N.  Amirdzhanova, A.E. Karateev, E.Yu. Pogozheva, E.S. Filatova, R.R. Samigullina, V.I. Mazurov, O.N. Anoshenkova, N.A. Lapkina, A.A. Baranov, T.Yu. Grineva, A.M. Lila, and E.L. Nasonov, was originally published electronically in Springer-Link on October 13, 2023 without Open Access. After publication in volume 511, pages 180–186, the authors decided to make the article an Open Access publication. Therefore, the copyright of the article has been changed to © The Author(s) 2023 and the article is forthwith distributed under the terms of a Creative Commons Attribution 4.0 International License (http://creativecommons.org/licenses/by/4.0/, CC BY), which permits use, duplication, adaptation, distribution and reproduction of a work in any medium or format, as long as you cite the original author(s) and publication source, provide a link to the Creative Commons license, and indicate if changes were made.

